# Supplementary material for: The Evolution and Characterization of the RNA Interference Pathways in Lophotrochozoa
Source: Genome Biol Evol. 2024 May 7;16(5):evae098. doi: 10.1093/gbe/evae098 (PMC11114477; doi:10.1093/gbe/evae098)
Supplement: evae098_Supplementary_Data [file evae098_supplementary_data.zip › Supplementary_materials.zip!/selected_miRNAs/NoGe_selected_miRNAs/mirdeep_pdf_files/NMRB01000004.1_Notospermus_geniculatus_strain_Ushimado_scaffold4,_whole_genome_shotgun_sequence_144.pdf]

Provisional ID	:	NMRB01000004.1_Notospermus_geniculatus_strain_Ushimado_scaffold4,_whole_genome_shotgun_sequence_144
Score total	:	456.3
Score for star read(s)	:	3.9
Score for read counts	:	451.3
Score for mfe	:	1.7
Score for randfold	:	0
Score for cons. seed	:	-0.6
Total read count	:	897
Mature read count	:	886
Loop read count	:	0
Star read count	:	11

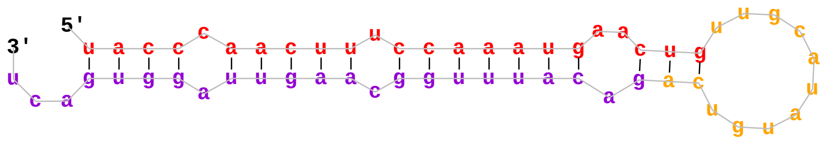

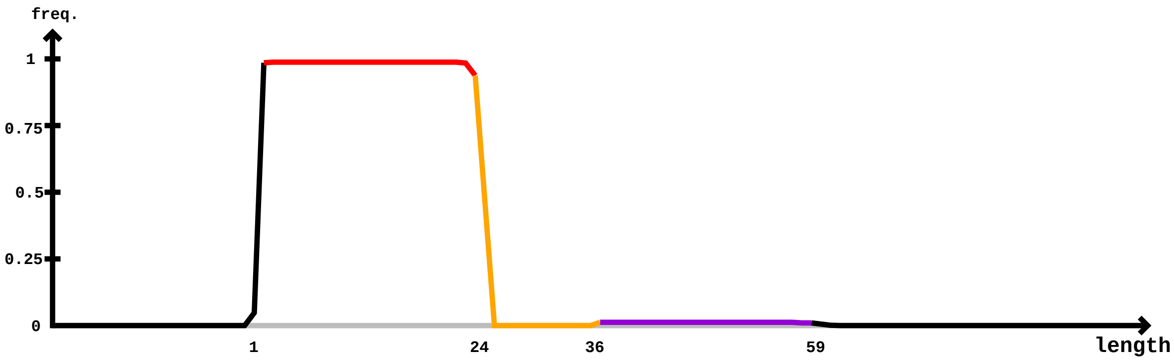

Mature Star

```
5'- ucacauuugccuagagcaguuacccaacuuuccaaaugaacuguugcauaugucagacauuuggcaaguuaggugacuguuccaggauugaaauuucugacaguaggggccu  -3'   obs
    ucacauuugccuagagcaguuacccaacuuuccaaaugaacuguugcauaugucagacauuuggcaaguuaggugacuguuccaggauugaaauuucugacaguaggggccu         exp
    ........((((.(((((((((((.(((((.(((((((..(((..........))).))))))).))))).)))))))))))(((((.......))))).......))))..   reads   mm   sample
    ...................uuacccaacuuuccaaaugaacu......................................................................   1       0    Mm3
    ...................uuacccaacuuuccaaaugaacug.....................................................................   8       0    Mm3
    ....................uacccaacuuuccaaaugaacu......................................................................   4       0    Mm3
    ....................Cacccaacuuuccaaaugaacug.....................................................................   1       1    Mm3
    ....................uacccaacuuuccaaaugaacug.....................................................................   29      0    Mm3
    ....................uacccaacuuAccaaaugaacug.....................................................................   1       1    Mm3
    ....................uacccaacuuuccaaaugaacugu....................................................................   1       0    Mm3
    .......................................................gacauuuggcaaguuagguga....................................   2       0    Mm3
    .......................................................gacauuuggcaaguuaggugacu..................................   1       0    Mm3
    .......................................................gacauuuggcaaguuaggugacuUu................................   1       1    Mm3

    ...................uuacccaacuuuccaaaCgaacug.....................................................................   1       1    Fm3
    ...................uuacccaacuuuccaaaAgaacug.....................................................................   1       1    Fm3
    ...................uuacccaacuuuccaaaugaacug.....................................................................   9       0    Fm3
    ....................uacccaacuuuccaaaugaac.......................................................................   1       0    Fm3
    ....................uacccaacuuuccaaaugaacu......................................................................   8       0    Fm3
    ....................uUcccaacuuuccaaaugaacug.....................................................................   1       1    Fm3
    ....................uacccGacuuuccaaaugaacug.....................................................................   1       1    Fm3
    ....................uaAccaacuuuccaaaugaacug.....................................................................   1       1    Fm3
    ....................uacccaacCuuccaaaugaacug.....................................................................   1       1    Fm3
    ....................uacccaacuuuccaaaugaacug.....................................................................   231     0    Fm3
    ....................uacccaacuuuccaaaGgaacug.....................................................................   1       1    Fm3
    ....................uacccaacuuuccaaauCaacug.....................................................................   1       1    Fm3
    ....................uacccaacuuuccaaaugaacugu....................................................................   4       0    Fm3
    .....................acccaacuuuccaaaugaacug.....................................................................   1       0    Fm3

    ....................uacccaacuuuccaaaugaacu......................................................................   6       0    Mm1
    ....................uacccaacuuuccaaaCgaacug.....................................................................   1       1    Mm1
    ....................uacccaacuuuccaaaugaacug.....................................................................   88      0    Mm1
    ....................uacccaacuuucUaaaugaacug.....................................................................   1       1    Mm1
    ....................uacAcaacuuuccaaaugaacug.....................................................................   1       1    Mm1

    ...................uuacccaacuuuccaaaugaacu......................................................................   2       0    Mm2
```

```
                   Mature                            Star
ucacauuugccuagagcaguuacccaacuuuccaaaugaacuguugcauaugucagacauuuggcaaguuaggugacuguuccaggauugaaauuucugacaguaggggccu
...................uuacccaacuuuccaaUugaacug.....................................................................    1    1    Mm2
...................uuacccaacuuuccaaauCaacug.....................................................................    1    1    Mm2
...................uuacccaacuuuccaaaugaacug.....................................................................    4    0    Mm2
....................uacccaacuuuccaaauCaac.......................................................................    1    1    Mm2
....................uacccaacuuuccaaaugaac.......................................................................    1    0    Mm2
....................uacccaacuuuccaaauCaacu......................................................................    7    1    Mm2
....................uacccaacuuuccaaaugaacu......................................................................    14   0    Mm2
....................uacccaacuuuccaaauAaacug.....................................................................    2    1    Mm2
....................uacccaacuuucUaaaugaacug.....................................................................    1    1    Mm2
....................uacccaacuuGccaaaugaacug.....................................................................    1    1    Mm2
....................uacccaacuuuccaaaAgaacug.....................................................................    1    1    Mm2
....................uacccaacuuuUcaaaugaacug.....................................................................    1    1    Mm2
....................uacccGacuuuccaaaugaacug.....................................................................    1    1    Mm2
....................uacccaacuuuccaaauCaacug.....................................................................    3    1    Mm2
....................uacccaacuuuccaaaugUacug.....................................................................    1    1    Mm2
....................uacccaacGuuccaaaugaacug.....................................................................    1    1    Mm2
....................uacccaacuuuccaaaCgaacug.....................................................................    4    1    Mm2
....................uGcccaacuuuccaaaugaacug.....................................................................    3    1    Mm2
....................uacccaacuuuccaaauUaacug.....................................................................    2    1    Mm2
....................uacccaacuuuccaGaugaacug.....................................................................    1    1    Mm2
....................uUcccaacuuuccaaaugaacug.....................................................................    1    1    Mm2
....................uaccAaacuuuccaaaugaacug.....................................................................    2    1    Mm2
....................uacccaacuuuccaaaugaacug.....................................................................    352  0    Mm2
....................uacccaacuuuccaaaugaacugu....................................................................    2    0    Mm2
.....................acccaacuuuccaaaugaacug.....................................................................    1    0    Mm2

...................uuacccaacuuuccaaaugaacug.....................................................................    15   0    Fm2
....................uacccaacuuuccaaaGgaacug.....................................................................    1    1    Fm2
....................uacccaacuuuccaaaugaacuU.....................................................................    1    1    Fm2
....................uacccaacuuuccaaauAaacug.....................................................................    1    1    Fm2
....................uGcccaacuuuccaaaugaacug.....................................................................    1    1    Fm2
....................uacccaacuuuccaaaugaacug.....................................................................    29   0    Fm2
....................uacccaacuuuccaaauCaacug.....................................................................    1    1    Fm2
.......................................................gacauuuggcaaguuaggugacu..................................    7    0    Fm2

....................uacccaacuuuccaaaugaacug.....................................................................    22   0    Fm1
```
